# Butein and Frondoside-A Combination Exhibits Additive Anti-Cancer Effects on Tumor Cell Viability, Colony Growth, and Invasion and Synergism on Endothelial Cell Migration

**DOI:** 10.3390/ijms23010431

**Published:** 2021-12-31

**Authors:** Shahrazad Sulaiman, Kholoud Arafat, Aya Mudhafar Al-Azawi, Noura Abdulraouf AlMarzooqi, Shamsa Nasser Ali Hussain Lootah, Samir Attoub

**Affiliations:** 1 Department of Pharmacology & Therapeutics, College of Medicine & Health Sciences, United Arab Emirates University, Al Ain 17666, United Arab Emirates; sharazadjeffy@uaeu.ac.ae (S.S.); kholoud.arafat@uaeu.ac.ae (K.A.); 201870001@uaeu.ac.ae (A.M.A.-A.); 201311678@uaeu.ac.ae (N.A.A.); 201312535@uaeu.ac.ae (S.N.A.H.L.); 2 Institut National de la Santé et de la Recherche Médicale (INSERM), 75013 Paris, France

**Keywords:** lung cancer, breast cancer, butein, frondoside-A, STAT3, angiogenesis, invasion, viability, tumor growth

## Abstract

Despite the significant advances in targeted- and immuno-therapies, lung and breast cancer are at the top list of cancer incidence and mortality worldwide as of 2020. Combination therapy consisting of a mixture of different drugs taken at once is currently the main approach in cancer management. Natural compounds are extensively investigated for their promising anti-cancer potential. This study explored the anti-cancer potential of butein, a biologically active flavonoid, on two major solid tumors, namely, A549 lung and MDA-MB-231 breast cancer cells alone and in combination with another natural anti-cancer compound, frondoside-A. We demonstrated that butein decreases A549 and MDA-MB-231 cancer cell viability and colony growth in vitro in addition to tumor growth on chick embryo chorioallantoic membrane (CAM) in vivo without inducing any noticeable toxicity. Additionally, non-toxic concentrations of butein significantly reduced the migration and invasion of both cell lines, suggesting its potential anti-metastatic effect. We showed that butein anti-cancer effects are due, at least in part, to a potent inhibition of STAT3 phosphorylation, leading to PARP cleavage and consequently cell death. Moreover, we demonstrated that combining butein with frondoside-A leads to additive effects on inhibiting A549 and MDA-MB-231 cellular viability, induction of caspase 3/7 activity, inhibition of colony growth, and inhibition of cellular migration and invasion. This combination reached a synergistic effect on the inhibition of HUVECs migration in vitro. Collectively, this study provides sufficient rationale to further carry out animal studies to confirm the relevance of these compounds’ combination in cancer therapy.

## 1. Introduction

Lung and breast cancer are the most prevalent types of cancer worldwide [1], and this is despite the significant advances in cytotoxic-, targeted-, and immune-therapies. While these treatment approaches have led to an improvement in overall survival, they come with drawbacks limiting their success in providing a cure for patients. Challenges of co-lateral damage, acquired resistance, and limited efficacy to a small percentage of patients, in addition to high cost, have led to a renewed interest in natural compounds for cancer treatment [2,3,4]. These natural compounds may have fewer side effects and may be used as an adjuvant to reduce clinical cytotoxic drug dose or minimize their side effects [5]. More importantly, they may also lead to a better anti-cancer response when used in combination therapy.

Extensive pre-clinical research studies are investigating natural compounds for their potential anti-cancer effects. Butein (3, 4, 2′, 4′-tetrahydroxychalcone) is one of these active ingredients, isolated from various plants including stem bark of cashews (*Semecarpus anacardium*), Rhus verniciflua Stokes, Caragana jubata, and the heartwood of Dalbergia odorifera [6]. The anti-cancer potential of butein has been demonstrated in leukemia and in various solid tumors investigated to date [6,7]. However, very few studies have investigated its anti-cancer effect in vivo or in combination with other natural agents such as frondoside-A, a triterpenoid glycoside isolated from the Atlantic cucumber, Cucumaria frondosa [8].

In this study, we determined the anti-cancer potential of butein on lung and breast cancer cell viability, colony growth, migration, and invasion in vitro and on tumor growth in vivo using the chick embryo CAM tumor xenograft model. The impact on angiogenesis was determined using HUVECs migration and tubules formation assays in vitro. In addition, the study investigated the anti-cancer impact of the combination of butein with the natural compound frondoside-A.

## 2. Results and Discussion

### 2.1. Butein Decreases Cellular Viability, Colony and Tumor Growth

**Figure 1:**
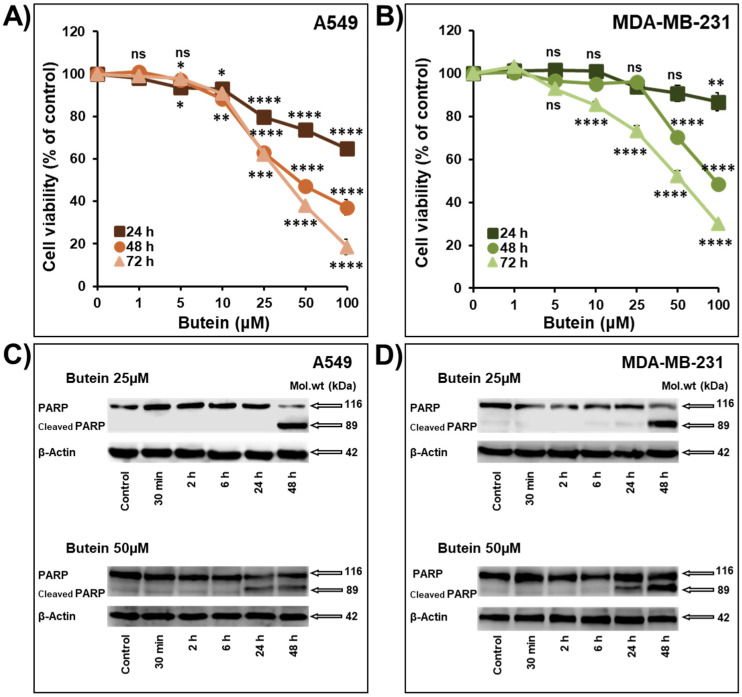
Inhibition of cellular viability associated PARP cleavage by butein. Exponentially growing A549 (**A**) and MDA-MB-231 (**B**) cells were treated with vehicle (0.1% DMSO) and the indicated concentrations of butein for 24, 48, and 72 h. Viable cells were determined using the CellTiter-Glo Luminescent Cell Viability Assay, based on ATP quantification, which indicates the presence of viable cells. Experiments were repeated at least three times. Western blot analysis shows PARP cleavage after butein (25 and 50 μM) treatment in A549 (**C**) and MDA-MB-231 (**D**) cancer cells. β-actin was used as a loading control. The data shown are representative of three in-dependent experiments. Shapes represent means; bars represent S.E.M. * Significantly different at *p* < 0.05, ** Significantly different at *p* < 0.01, *** Significantly different at *p* < 0.001, **** Significantly different at *p* < 0.0001. ns—non-significant.

The anti-cancer effect of butein was investigated on cell viability, colony and tumor growth in non-small cell lung cancer (NSCLC) cells A549 and triple-negative breast cancer (TNBC) cells MDA-MB-231. As shown in Figure 1, butein (1–100 µM) caused a concentration- and time-dependent decrease in the cellular viability of lung A549 and breast MDA-MB-231 cells (Figure 1A,B). The IC50 values at 72 h were 35.1 µM and 55.7 µM for A549 and MDA-MB-231 cells, respectively. Our results are in agreement with other studies showing that butein suppressed the survival of multiple myeloma cells U266 [9], prostate cancer cells “LNCaP, CWR22Rv1, and PC-3” [10], NSCLC cells A549 [11], cervical cells HeLa [12], C-33A and SiHa [13], breast cancer cells ER+ “MCF7, T47D, and ZR-75-1” and the TNBC cells “MDA-MB-231, Hs578T, BT-20, HCC-38, HCC-70 and MDA-MB-453” [14,15], hepatocarcinoma cells SK-HEP-1 [16], and acute lymphoblastic leukemia cells “RS4-11, CEM-C7, CEM-C1, and MOLT-4” [17].

Cleavage of PARP is widely accepted as a specific marker of apoptosis. In this study, we clearly demonstrated that butein (25 and 50 μM) induces a concentration- and time-dependent increase in the cleavage of the full-length PARP (116 kDa) to a large cleaved fragment (89 kDa) in both A549 and MDA-MB-231 cells (Figure 1C,D). These data are supported by previous reports demonstrating that 24 h treatment with butein induces PARP cleavage in HepG2 cells [18,19], LNCaP cells [10], and C-33A and SiHa [13].

**Figure 2:**
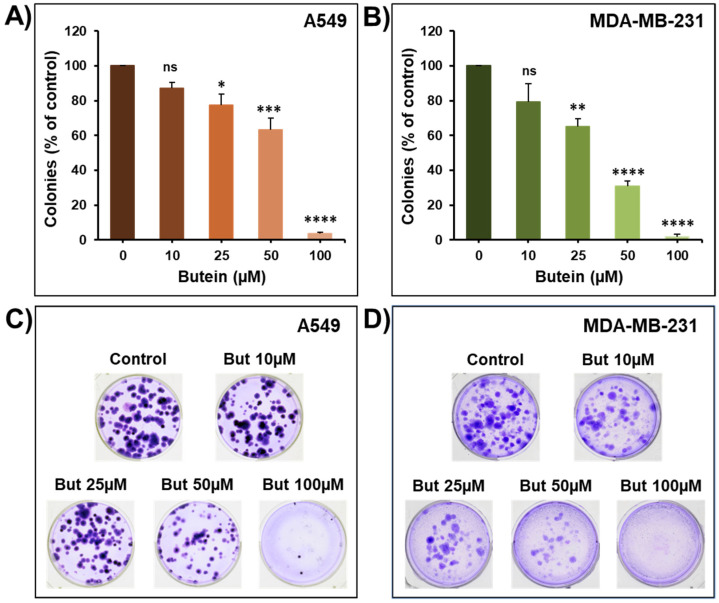
Effect of butein on colony growth. The growth of cancer cell-derived colonies from A549 (**A**) and MDA-MB-231 (**B**) cells was assessed by measuring the number of the colonies in control and butein-treated wells for seven days. (**C**,**D**) Representative pictures of the control and butein-treated colonies are shown for A549 and MDA-MB-231 cancer cells. Experiments were repeated at least three times. Columns represent means; bars represent S.E.M. * Significantly different at *p* < 0.05, ** Significantly different at *p* < 0.01, *** Significantly different at *p* < 0.001, **** Significantly different at *p* < 0.0001. ns—non-significant.

To further confirm the anticancer potential of butein, we examined its ability to affect the growth capacity of pre-formed colonies using the colony growth assay. Toward this, lung (A549) and breast (MDA-MB-231) cells were grown for 14 days to form colonies and then treated with increasing concentration of butein for an additional seven days. Treatment with butein (10–100 µM) caused a significant concentration-dependent decrease in the number of colonies (Figure 2A–D). Similarly, it has been shown that butein decreases the colony formation of HeLa cells [12], and SMMC-7721 and HepG2 hepatocarcinoma cells [19].

**Figure 3:**
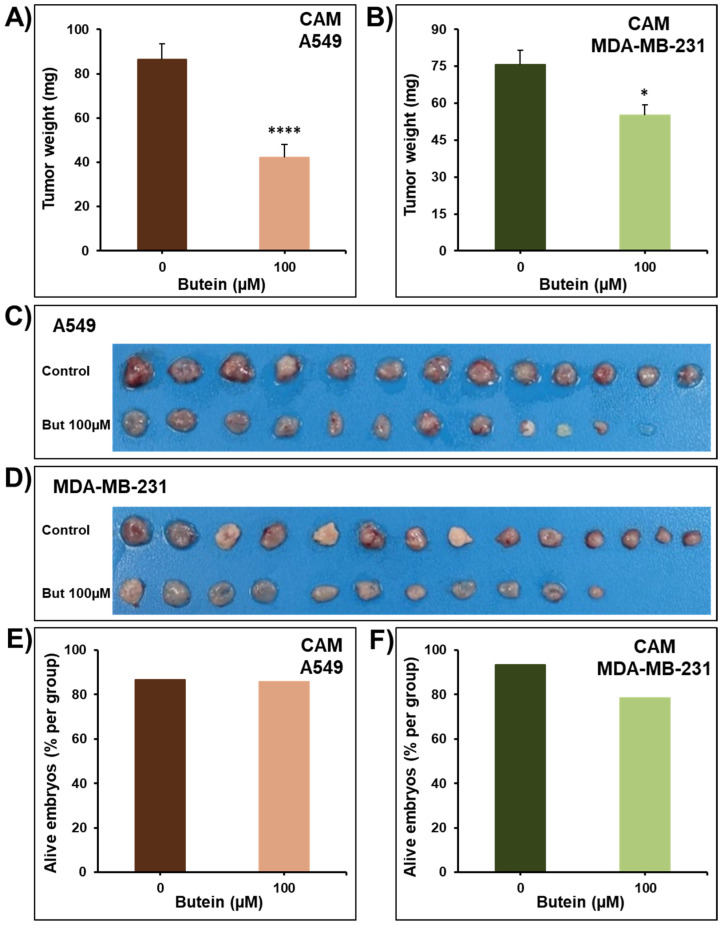
Impact of butein on tumor growth using the in vivo CAM tumor xenograft model. Volumes of 1 × 10^6^ of A549 (**A**,**C**) and MDA-MB-231 (**B,D**) cells were grafted on the CAM of 9 days (E9) chick embryos. Tumors were treated with butein (100 µM) every 48 h for a total of 6 days. At E17, tumors were collected, weighed, and photographed (**C**,**D**). The viability of the chick embryos was assessed, and the percentage of alive embryos was determined (**E**,**F**). Columns are means; bars are S.E.M. * Significantly different at *p* < 0.05. **** Significantly different at *p* < 0.0001.

To confirm the pharmacological relevance of our in vitro data, the anticancer activity of butein was investigated in vivo using the CAM tumor growth model. A549 and MDA-MB-231 cells grafted on the CAM formed tumors that were treated every 48 h with vehicle (DMSO) or butein (100 µM). At the end of the experiment (E17), tumors were recovered from the upper CAM and weighted. In line with our in vitro findings, we found that butein significantly inhibited tumor growth in vivo (Figure 3A–D). Butein showed no major toxic effect as there was no, or a small, difference in the number of surviving chick embryos between the control and the butein-treated eggs in A549 groups (85.7% vs. 86.7%) (Figure 3E) and MDA-MB-231 groups (78.6% vs. 93.3%) (Figure 3F). Similarly, it has been reported that butein decreases cervical HeLa [12], breast HER2^+^ BT-474 [15], and liver HepG2 [19,20] xenografts tumor growth in nude mice.

### 2.2. Butein Decreases Lung and Breast Cancer Cell Migration and Invasion

**Figure 4:**
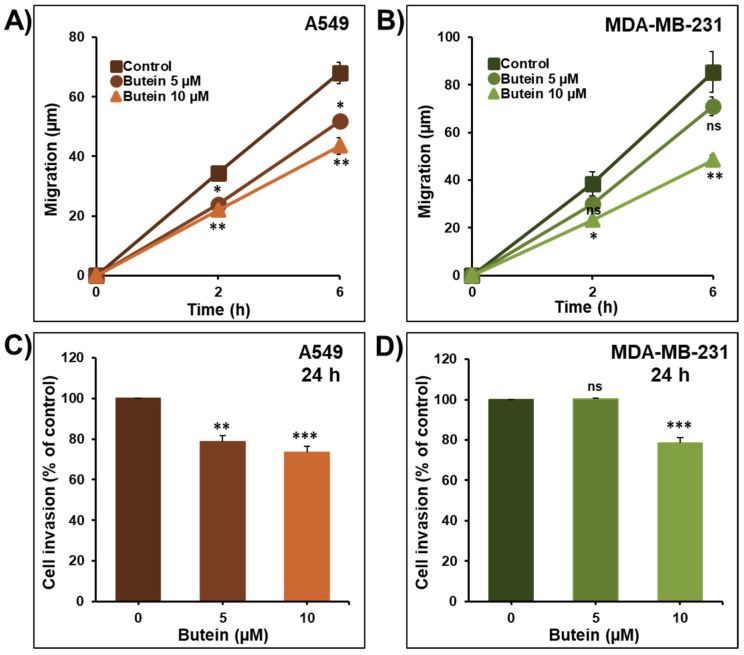
Butein impairs cancer cell migration and invasion. Wounds were introduced in A549 (**A**) and MDA-MB-231 (**B**) cells’ confluent monolayers cultured in the presence or absence (control) of butein (5 and 10 µM). The mean distance that cells travelled from the edge of the scraped area after 2 and 6 h was measured using an inverted microscope. A549 (**C**) and MDA-MB-231 (**D**) cells were incubated for 24 h in the presence or absence of butein (5 and 10 µM). Cells that invaded the Matrigel and crossed the 8 µm pores insert were determined using the CellTiter-Glo Luminescent Cell Viability Assay. All experiments were repeated at least three times. Columns or shapes represent means; bars represent S.E.M. * Significantly different at *p* < 0.05, ** Significantly different at *p* < 0.01, *** Significantly different at *p* < 0.001. ns—non-significant.

Cancer cell migration and invasion are critical steps in the process of metastasis. To determine whether butein inhibited cell migration and invasion in vitro, lung A549 and breast MDA-MB-231 cancer cells were treated with low concentrations of butein (5 and 10 µM). As shown in Figure 4, butein induced a significant time- and concentration-dependent inhibition of A549 (Figure 4A) and MDA-MB-231 (Figure 4B) cancer cell migration. In addition, we demonstrated that butein was also able to decrease A549 and MDA-MB-231 cancer cell invasion in a concentration-dependent manner (Figure 4C,D).

A previous study also reported that butein at the concentration of 20 µM suppressed bladder cancer cells BLS-211 motility and invasion [20]. Similarly, butein at the high concentration of 50 µM suppressed CXCL12-induced cancer cell migration and invasion of breast cancer cells SKBr3 and the pancreatic cells AsPC-1 [21], respectively. Butein (15, 25, and 50 µM) also decreased HeLa cell migration and invasion [12], and at the concentrations of 50 and 75 µM inhibited the migration and invasion of SK-HEP-1 cells [16]. In comparison with our study, all previous studies used a much higher concentration of butein. In addition, it has been shown that butein reduces lung metastasis of mouse melanoma cells B16F10 [22] and the metastasis behavior of the hepatocellular carcinoma cells SK-Hep-1 [23].

### 2.3. Impact of Butein on STAT3 Phosphorylation

**Figure 5:**
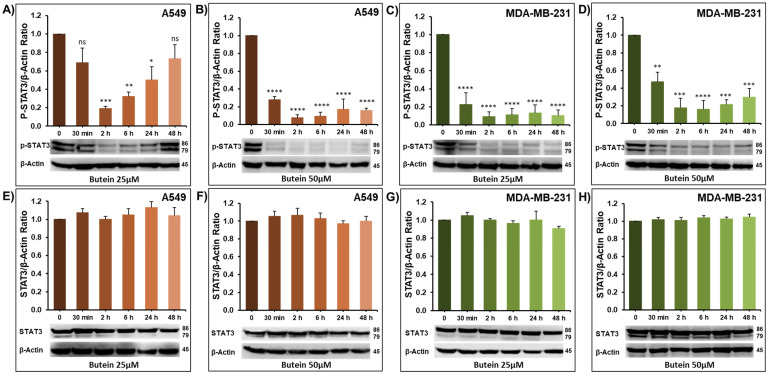
Western blot showing the inhibition of STAT3 phosphorylation by butein in A549 lung cancer cells (**A**,**B**), and MDA-MB-231 breast cancer cells (**C**,**D**). Effect of butein on total STAT3 (**E**–**H**). Each cell line was treated with 25 and 50 μM butein, and proteins were extracted at the indicated time-points (0.5, 2, 6, 24, and 48 h). β-actin was used as a loading control. The data shown are representative of three independent experiments. Columns represent means; bars represent S.E.M. * Significantly different at *p* < 0.05, ** Significantly different at *p* < 0.01, *** Significantly different at *p* < 0.001, **** Significantly different at *p* < 0.0001. ns—non-significant.

Signal Transducer and Activator of Transcription 3 (STAT3) is constitutively active in a wide variety of human cancers, including but not limited to breast, lung, and colorectal cancers [24,25]. Chronic STAT3 phosphorylation is associated with major cancer hallmarks, including survival, migration, invasion, and metastasis [26,27,28,29]. There is large community agreement that the activated STAT3 influences not only tumor growth but also the invasiveness of cancer cells [30]. However, STAT3 targeting for cancer therapy is still a major challenge [24,31,32,33]. In this context, we examined whether the observed anti-cancer effects of butein involve the STAT3 pathway. In this sense, the level of activated (phosphorylated) STAT3 was examined over time-period (0.5, 2, 6, 24, and 48 h) in the lung (A549) and breast (MDA-MB-231) cancer cells lines treated with 25 and 50 μM of butein. As shown in Figure 5, butein significantly decreased the level of phosphorylated STAT3 in A549 (Figure 5A,B) and MDA-MB-231 (Figure 5C,D). The inhibition of STAT3 phosphorylation was observed as early as 30 min post-treatment at both used concentrations and in both cell lines. This inhibition was maintained for almost 48 h. As expected, butein treatment had no effect on the level of total STAT3 (Figure 5E–H). Our results are in agreement with previous reports showing that butein inhibited the constitutive activation of STAT3 in multiple myeloma cells U266 [9] and in the hepatocellular carcinoma cell line HepG2 [18]. These two studies also demonstrate that this suppression of STAT3 phosphorylation was mediated through the inhibition of the upstream-activated c-src and JAK2 kinases. Butein also inhibited the constitutive active STAT3 in both head and neck squamous carcinoma SCC4 and in human prostate carcinoma DU145 cells [9]. Altogether, these data strongly suggest that butein mediates its anti-cancer effect, at least in part, through downregulation of the STAT3 signaling pathway.

### 2.4. Butein in Combination Therapy with Frondoside-A Enhances Caspase 3/7 Inhibition of Cellular Viability

Frondoside-A has been previously reported to have strong anticancer activity against various types of cancer, including breast, lung, pancreas, prostate, and colon cancer [34,35,36,37,38,39,40]. The widespread effects of frondoside-A have been linked to various mechanisms, notably, inhibition of P21-activated kinase 1 (PAK1) [38,41]. Nguyen et al. (2017) reported that frondoside-A directly and specifically inhibits PAK1 activity in vitro with an IC50 equal to 1.2 µM [41]. PAK1 is overexpressed or overactivated in various cancer types, controlling cell growth, autophagy, angiogenesis, invasion, and metastasis [42]. Therefore, inhibiting such targets by frondoside-A could explain the wide range of activities frondoside-A exerts [38]. In current clinical oncology practices, combination therapy is the main approach in cancer management [43]. Therefore, we decided to explore the anticancer activity of butein combined with frondoside-A.

To investigate the therapeutic value of combining butein with frondoside-A, we used concentrations of butein (50 μM) and frondoside-A (2.5 μM) that induced a 50% decrease in cell viability of A549 cells at 48 h. However, for MDA-MB-231 cells, we combined concentrations of butein (50 μM) and frondoside-A (1 μM) that induced an almost 25% decrease in cell viability at 48 h.

**Figure 6:**
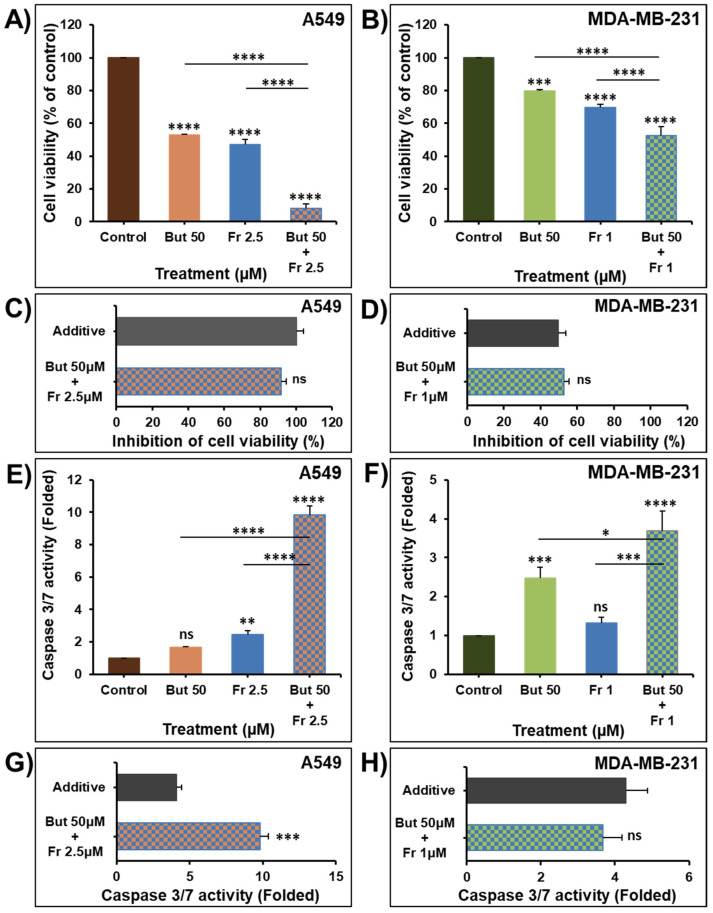
Effect of butein in combination with frondoside-A on the inhibition of cell viability of A549 (**A**) and MDA-MB-231 cells (**B**) after 48 h treatment. Effect of combinations of butein and frondoside-A on cell viability compared with the calculated additive effects of the two drugs alone (**C**,**D**). Induction of caspase 3/7 activity was also analyzed in A549 and MDA-MB-231 cells treated for 48 h with frondoside-A (2.5 and 1 µM, respectively), butein (50 µM) and their combination (**E**,**F**). Effect of combinations of butein and frondoside-A on caspase 3/7 activity compared with the calculated additive effects of the two drugs alone (**G**,**H**). Data were normalized to the number of viable cells per well and expressed as fold induction compared to the control group. All experiments were repeated at least three times. Columns are means; bars are S.E.M. The statistical significance is compared to the control except for the specified lines. * Significantly different at *p* < 0.05, ** Significantly different at *p* < 0.01, *** Significantly different at *p* < 0.001, **** Significantly different at *p* < 0.0001. ns—non-significant.

We demonstrated that treatment of the A549 and MDA-MB-231 cells for 48 h with frondoside-A (1 and 2.5 μM) significantly enhance the inhibitory effects of butein 50 μM on cell viability (Figure 6A,B). This combination produced an inhibition of cell viability equal to the calculated additive effects of the drugs used alone (Figure 6C,D), demonstrating a clear additive effect between butein and frondoside-A in the inhibition of cellular viability.

Caspase 3 activation induces the cleavage and consequently the inactivation of the downstream PARP events, leading to apoptosis [37]. The treatment of A549 and MDA-MB-231 cancer cells with butein (50 μM) for 48 h induced around two-fold increase in caspase 3/7 activity (Figure 6E,F). These data are in agreement with previous studies showing that treatment with butein increased caspase 3, 8, and 9 activities in HeLa cells [12], LNCaP cells [10], SKOV-3/PAX ovarian cancer cells [44], and cervical cancer cells C-33A and SiHa [13].

Despite the mild induction in caspase 3/7 activity when used alone, butein significantly synergizes with frondoside-A (2.5 μM) in the activation of caspase 3/7 in A549 cells (Figure 6E,G). In MDA-MB-231 cells, 48 h combination of butein (50 μM) and frondoside-A (1 μM) produced an increase in caspase 3/7 activity equal to the calculated additive effect of the drugs used alone (Figure 6F,H). 

### 2.5. Impact of Butein in Combination with Frondoside-A on Colony Growth

**Figure 7:**
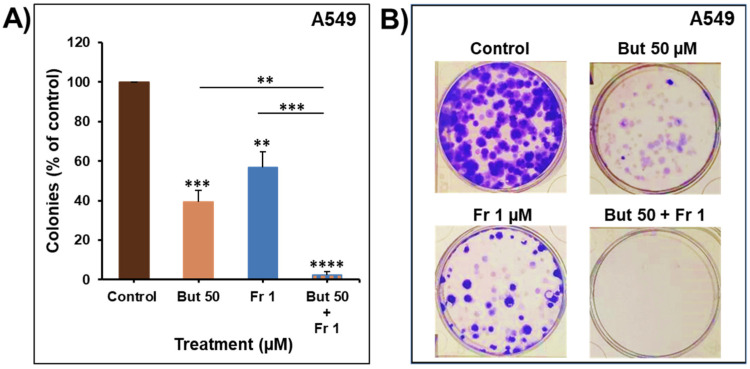
Impact of butein in combination with frondoside-A on A549 colony growth after 14 days of treatment (**A**,**B**). Experiments were repeated three times. Columns represent means; bars represent S.E.M. The statistical significance is compared to the control except for the specified lines. ** Significantly different at *p* < 0.01, *** Significantly different at *p* < 0.001, **** Significantly different at *p* < 0.0001.

To further assess the anti-cancer potential of combining butein with frondoside-A, the combined impact was investigated on the growth of pre-formed colonies of A549. Toward this, A549 cells were grown for ten days to form colonies that were treated with butein 50 μM, frondoside-A 1 μM, or a combination of both. As shown in Figure 7, combination treatment of the pre-formed colonies for two weeks significantly decreased the number of colonies compared to individual therapy. The combination caused additive effects by reducing the number of colonies by 98 ± 2%, similar to the calculated additive value of single treatments (104 ± 6%).

### 2.6. Impact of Butein in Combination of Frondoside-A on Angiogenesis In Vitro

**Figure 8:**
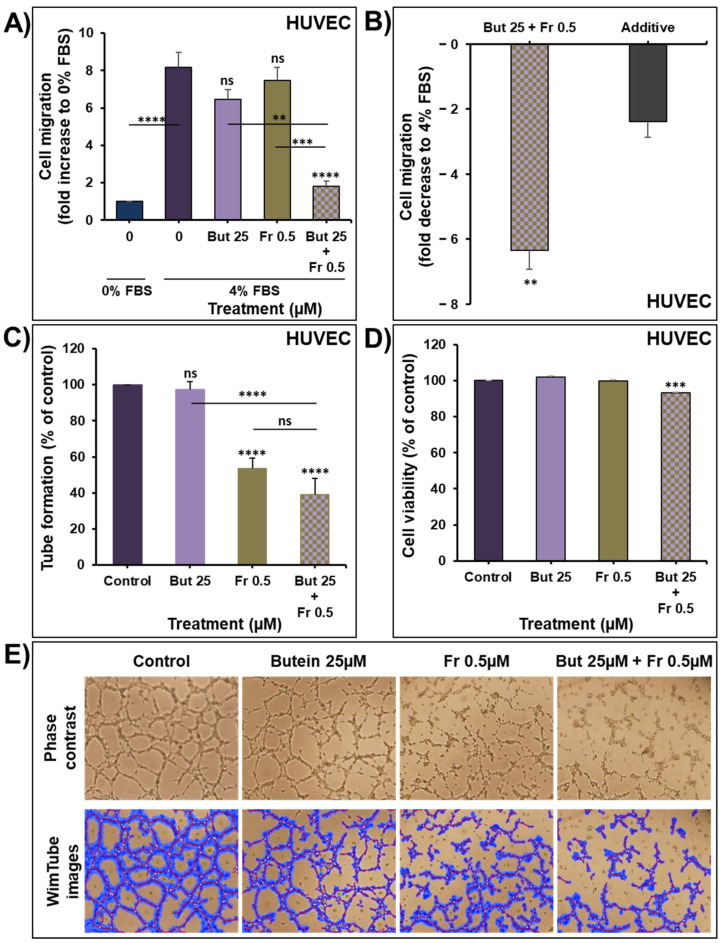
Effect of butein (25 µM) in combination with frondoside-A (0.5 µM) on HUVECs migration after 8 h of treatment (**A**). Effect of the combination on HUVECs migration compared with the calculated additive effects of the two drugs alone (**B**). Data were expressed as fold induction compared to the control group. (**C**–**E**) Impact of butein and frondoside-A alone and in combination on HUVECs capillary-like-structure formation and cell viability after 8 h of treatment. The statistical significance is compared to the control (4% FBS) except for the specified lines. All experiments were repeated at least three times. Columns are means; bars are S.E.M. ** Significantly different at *p* < 0.01, *** Significantly different at *p* < 0.001, **** Significantly different at *p* < 0.0001. ns—non-significant.

To further evaluate the therapeutic value of combining butein with frondoside-A, we investigated the impact of this combination on angiogenesis in vitro. First, the effect of the combination on HUVECs migration was determined for eight hours using transwell chambers. As shown in Figure 8A, 4% FBS increased HUVECs migration by eight-fold compared to 0% FBS. Treatment with butein (25 µM) or frondoside-A (0.5 µM) slightly decreased the HUVECs migration without reaching statistical significance. However, the combination of butein and frondoside-A led to a significant decrease in the FBS-induced HUVECs migration. This combination produced a more potent inhibition in the HUVECs migration than the calculated additive effects of the drugs used alone, demonstrating a clear synergism between butein and frondoside-A in the inhibition of HUVECs migration (Figure 8B).

The effect of this combination was next investigated on the ability of HUVECs to form capillary-like structures when seeded on Matrigel. As shown in Figure 8C–E, butein failed to inhibit the ability of HUVECs to form capillary-like structures, in contrast to frondoside-A, which significantly decreased the tube formation by approximately 40%. Combination of butein with frondoside-A slightly enhanced the frondoside-A inhibitory effect without reaching statistical significance. The impact of these treatments on HUVECs viability was determined at the end of the capillary-like-structure experiments. As you can see in Figure 8D, butein and frondoside-A did not affect HUVECs cell viability. However, their combination led to a slight, 7% decrease in cell viability (Figure 8D). Combining butein with frondoside-A could be a promising approach to target endothelial migration, an essential step in angiogenesis.

To the best of our knowledge, this is the first study demonstrating the synergistic impact of butein and frondoside-A on endothelial migration in vitro. The anti-angiogenic effect of butein in vitro was documented only once by Chung et al., (2013), who reported the ability of butein (1–20 µM) to inhibit the migration and tube formation of human endothelial progenitor cells in a concentration-dependent manner [45]. The differences in the endothelial cell type and experiment condition between the aforementioned report and this study might explain the variable results.

### 2.7. Additive Inhibition of Cellular Invasion by the Combination of Butein with Frondoside-A

**Figure 9:**
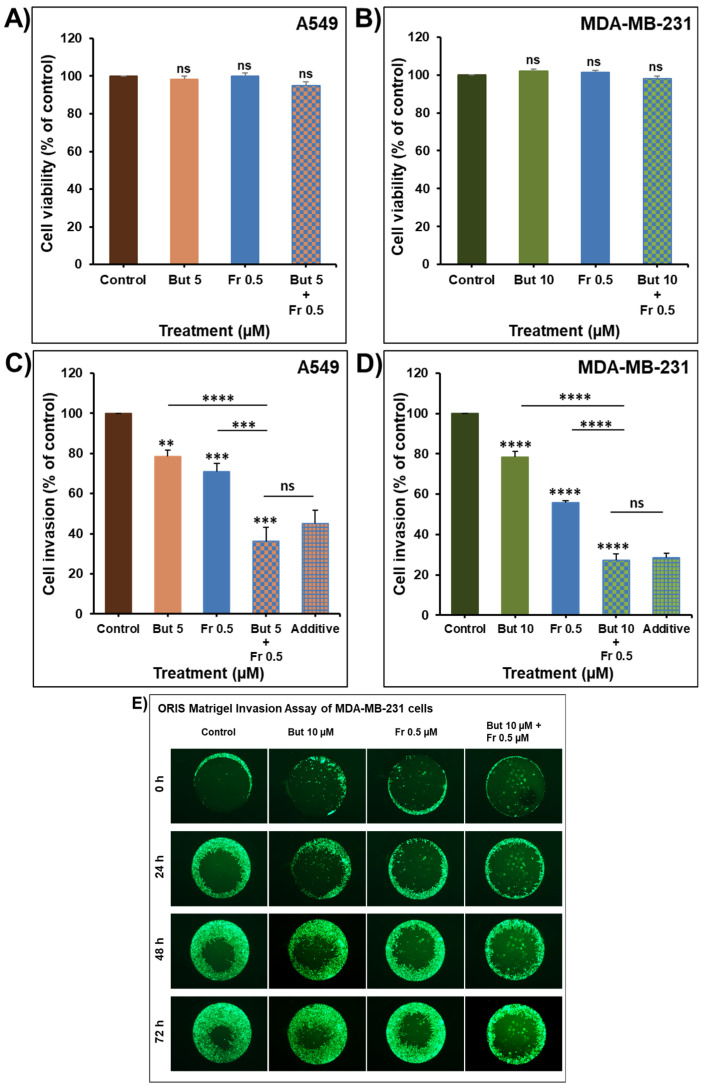
Impact of butein combination with frondoside-A on cellular invasion. A549 (**A**) and MDA-MB-231 (**B**) cells were treated for 24 h with butein (5 and 10 µM, respectively), frondoside-A (0.5 µM), and their combination. The effect on cell viability was determined as previously described. Using a Boyden chamber Matrigel invasion assay, A549 (**C**) and MDA-MB-231 (**D**) cells were incubated for 24 h with the non-toxic concentrations of butein and frondoside-A and their combination. Cells that invaded the Matrigel and crossed the 8 µm pores were determined using the CellTiter-Glo Luminescent Cell Viability Assay. (**E**) Oris Matrigel invasion assay showing an enhanced suppression of MDA-MB-231 cell invasion in the combination of butein with frondoside-A compared to drugs alone. All experiments were repeated at least three times. Columns are means; bars are S.E.M. The statistical significance is compared to the control except for the specified lines. ** Significantly different at *p* < 0.01, *** Significantly different at *p* < 0.001, **** Significantly different at *p* < 0.0001. ns–not significant.

To further evaluate the therapeutic value of combining butein with frondoside-A, we investigated whether the anti-invasive effect of butein could enhance the anti-invasive potential of frondoside-A. In this context, we have previously reported that frondoside-A possesses strong anti-invasiveness activity against breast [34] and lung [35] cancer cells. Treatment of A549 and MDA-MB-23 cells for 24 h with a low concentration of butein (5 and 10 µM, respectively) or frondoside-A (0.5 µM) (Figure 9A,B) led to a significant decrease in the invasiveness of the two cell lines using the Boyden chamber invasion assay. Similar to the cell viability data, the combination of butein and frondoside-A produced a decrease in cellular invasion of both cell lines equal to the calculated additive effects of the drugs used alone (Figure 9C,D). The anti-invasiveness additive effect of the combination of butein (10 µM) and frondoside-A (0.5 µM) for 24, 48, and 72 h was also confirmed in the ORIS Matrigel invasion assay using the MDA-MB-231 cells (Figure 9E).

## 3. Materials and Methods

### 3.1. Cell Culture and Reagents

Human NSCLC cells A549 were maintained in RPMI 1640 (Hyclone, Cramlington, UK), and human TNBC cells MDA-MB-231 were maintained in DMEM (Hyclone, Cramlington, UK). All media were supplemented with 1% of Penicillin-Streptomycin solution (Hyclone, Cramlington, UK) and with 10% fetal bovine serum (FBS; Hyclone, Cramlington, UK). Human Umbilical Vein Endothelial Cells (HUVECs) (Millipore, Temecula, CA, USA) were maintained in an EndoGRO^TM^-VEGF complete media kit (Millipore, Temecula, CA, USA) in flasks coated with 0.2% Gelatin. The culture medium of all cells was changed every 3 days, and cells were passed once a week when the culture reached 95% confluency for cancer cells and 80% for HUVECs. In all experiments, cell viability was higher than 99% using trypan blue dye exclusion. Butein and frondoside-A were purchased from Sigma-Aldrich (Sigma-Aldrich, Saint Louis, MO, USA).

### 3.2. Cellular Viability

Cells were seeded at a density of 5000 cells/well into 96-well plates. After 24 h, cells were treated for another 24, 48, and 72 h with increasing concentrations of Butein (1–100 µM) in triplicate. Control cultures were treated with 0.1% DMSO (the drug vehicle). The effect of butein on cell viability was determined using the CellTiter-Glo Luminescent Cell Viability Assay (Promega Corporation, Madison; US), based on quantification of ATP, which indicates the presence of metabolically viable cells. The luminescent signal was measured using the GLOMAX Luminometer (Promega Corporation, Madison, WI, USA). Cellular viability was presented as a percentage (%) by comparing the butein-treated cells with the DMSO-treated cells, the viability of which is assumed to be 100%.

In the second set of experiments, cells were treated for 48 h with a combination of butein (50 µM) and frondoside-A (1 and 2.5 µM). The effects of these combinations on cell viability were presented as proportional cell viability (%) by comparing the drugs-treated cells with the DMSO-treated cells, the viability of which is assumed to be 100%.

### 3.3. Caspase 3/7 Activity

Cells were seeded at a density of 5000 cells/well into 96-well plate and treated with butein (50 µM) and frondoside-A (1 and 2.5 µM) for 48 h, in triplicate. Control cells were exposed to DMSO 0.1%. Caspase 3/7 activity was measured using a luminescent Caspase-Glo 3/7 assay kit, following the manufacturer’s instructions (Promega Corporation, Madison, WI, USA). Caspase reagent was added, and the plate was mixed and incubated for 2.5 h at room temperature. Luminescence was measured using a GLOMAX Luminometer. Caspase 3/7 activity was normalized to the cellular viability and expressed as fold changes.

### 3.4. Clonogenic Assay

A549 and MDA-MB-231 cells were seeded into six-well plates at 100 cells/well. Cells were incubated for 14 days to form colonies and then treated every 3 days for another 7 days with increasing concentration of butein (10–100 µM). Colonies were then washed three times with PBS, fixed, and stained for 2 h with 0.5% crystal violet dissolved in (*v*/*v*) distilled water/methanol. Colonies were again washed three times with PBS, photographed, and counted. The percentages of colonies with more than 50 cells were determined and compared to the DMSO-treated colonies assumed to be 100%. The experiment was repeated three times. Data were presented as colonies percentage (%) by comparing the treated colonies with the control colonies. Colonies from representative experiments were photographed using an inverted phase-contrast microscope.

In the second set of experiments, A549 cells were kept for 7 days to form colonies and then were treated every 3 days for 14 days with a combination of butein (50 µM) and frondoside-A (1 µM). Data were presented as colonies percentage (%) by comparing the drug-treated colonies with the control colonies.

### 3.5. In Ovo Tumor Growth Assay

Fertilized White Leghorn eggs were incubated at 37.5 °C and 50% humidity. At the embryonic day 3 (E3), the CAM was dropped by drilling a small hole through the eggshell opposite to the round wide end followed by aspirating ~1.5–2 mL of albumin using a 5 mL syringe with an 18 G needle. Then, a small 1 cm^2^ window was cut in the eggshell above the CAM using a delicate scissor and sealed with a semipermeable adhesive film (Suprasorb^®^ F). At day 9 (E9), cancer cells were trypsinized, washed with complete medium, and suspended at a density of 1 × 10^6^ cells/100 µL in 80% Matrigel Matrix (Corning, Bedford, UK). A 100 µL inoculum of cell suspension was added onto the CAM of each egg, for a total of 14–15 eggs per condition. Two days later, tumors were treated topically every second day at E11, E13, and E15, by dropping 100 μL of the vehicle (PBS with 0.1% of DMSO) or butein (100 µM). At the embryonic day 16 (E16), embryos were humanely euthanized by topical addition of 10–30 µL of Pentobarbitone Sodium (300 mg/mL, Jurox, Auckland, New Zealand). Tumors were carefully extracted from the upper CAM tissues, washed with PBS, and weighted to determine the effect of butein on tumor growth. Drug toxicity was assessed by comparing the percentage of alive embryos in the control and butein-treated groups at the end of the experiment. Alive embryos were determined by checking the voluntary movements of the embryos in addition to the integrity and pulsation of the blood vessels. The eggs were randomly assigned to the treatments, but the experimenter was not blinded to the identities of the groups. All data collected were used in statistical analysis. This assay was carried out according to the protocol approved by the animal ethics committee at the United Arab Emirates University. According to the European Directive 2010/63/EU on the protection of animals used for scientific purposes, experiments involving using chicken embryos on and before E18 do not require approval from the Institutional Animal Care and Use Committee (IACUC).

### 3.6. Scratch Wound Healing Migration Assay

A549 and MDA-MB-231 cells seeded at a density of 1.75 × 10^6^ cells/well into a six-well plate reached confluence after 24 h. Then, a scrape was made through the confluent monolayer using a 200 µL tip. Afterwards, the dishes were washed twice and incubated at 37 °C in fresh medium containing 10% fetal bovine serum and two low concentrations of butein (5 and 10 µM). At the top side of each well, two random places were marked where the width of the wound was monitored using an inverted microscope at objective 4× (Olympus, Tokyo, Japan). Migration was expressed as the mean ± SEM of the wound difference between the measurements at time zero and the 2 and 6 h time-periods considered.

### 3.7. Boyden Chamber Matrigel Invasion Assay

The invasiveness of the lung cancer cells A549 and the breast cancer cells MDA-MB-231 was tested using a Corning BioCoat Matrigel Invasion Chamber (8 µm pore size) in a 24-well plate (Corning, Bedford, MA, USA), according to the manufacturer’s protocol. Cells (1 × 10^5^) in 0.5 mL of serum-free media were seeded into the upper chambers of the system with the indicated concentration of butein, frondoside-A, or butein in combination with frondoside-A. The bottom wells in the system were filled with the corresponding media supplemented with 10% fetal bovine serum as a chemoattractant and then incubated at 37 °C for 24 h. Non-invasive cells were removed from the upper surface of the filter by gently rubbing the area with a cotton swab. Cells that invaded the Matrigel and passed through the 8 µm pores of the insert were detected using CellTiter-Glo^®^ Luminescent Cell Viability assay (Promega Corporation, Madison, WI, USA). This was done by incubating the inserts into wells having CellTiter-Glo^®^ reagent mixed with medium (1:1) for 10 min, after which the luminescence signal was measured as described in the cellular viability section. The effects of the treatments on cellular invasion were presented as a percentage (%) by comparing the invading cells in the presence of the treatments with the control condition.

### 3.8. The Oris™ Matrigel Cell Invasion Assay

The impact of butein and frondoside-A, respectively, compared to the combination butein/frondoside-A on the invasiveness of MDA-MB-231-GFP cells was also investigated using a three-dimensional extracellular Matrigel matrix (Corning, Bedford, UK). Cells were seeded at 100,000 cells/well and allowed to attach overnight onto a 96-well plate coated with Matrigel. Once the cells formed a confluent monolayer, the silicone stoppers were removed. Wells were washed twice with PBS and then the cells were covered with 40 µL of Matrigel at the concentration of 6 mg/mL, incubated at 37 °C in the incubator for 45 min, and then incubated in complete media with the indicated treatments for 24, 48, and 72 h. The impact of the treatments of the invasiveness of the MDA-MB-231 GFP cells was assessed using an Olympus fluorescence microscope (Olympus, Tokyo, Japan). Representative figures were taken at 0, 24, 48, and 72 h.

### 3.9. Western Blotting Assay

A549 and MDA-MB-231 cells were seeded in 60 mm dishes at 750,000 cells/dish for 24 h and then treated with two concentrations of butein (25 and 50 µM) for another 0.5, 2, 6, 24, and 48 h. Control cultures were treated with 0.1% DMSO (the drug vehicle). Total cellular proteins were isolated using RIPA buffer (25 mM Tris.HCl, pH 7.6; 1% Nonidet P-40; 1% sodium deoxycholate; 0.1% SDS; 0.5% protease inhibitor cocktail; 1% PMSF; 1% phosphatase inhibitor cocktail) from the DMSO- and drug-treated cells. The whole-cell lysates were recovered by centrifugation at 14,000 rpm for 20 min at 4 °C to remove insoluble material, and protein concentrations of lysates were determined using a BCA protein assay kit (Thermo Fisher Scientific, Waltham, MA, USA). Proteins (30 µg) were separated by SDS-PAGE gel to determine the expression of STAT3, the level of p-STAT3, and PARP cleavage. After electrophoresis, the proteins were transferred onto a nitrocellulose membrane, blocked for 1 h at room temperature with 5% non-fat milk in TBST (TBS and 0.05% Tween 20), and then probed with specific primary antibodies and β-actin overnight at 4 °C. Antibodies to STAT3 (124H6) (1:1000), phospho-STAT3 (Tyr705) (D3A7) XP^®^ (1:600), and cleaved poly (ADP-ribose) polymerase (PARP) (1:500) were obtained from Cell Signaling Technology (Cell Signaling, Beverly, MA, USA). The β-actin antibody (1:9000) was obtained from Santa Cruz Biotechnology, Inc (Santa Cruz, CA, USA). Blots were washed and exposed to secondary antibodies. Immunoreactive bands were detected using ECL substrate (Thermo Fisher Scientific, Waltham, MA, USA) and chemiluminescence was detected using the LiCOR C-DiGit blot scanner (LI-COR Biotechnology, Lincoln, NE, USA). Densitometry analysis was performed using an HP Deskjet F4180 Scanner with ImageJ software. The intensities of the bands were normalized to the intensities of the corresponding β-actin bands.

### 3.10. HUVECs Migration Assay

HUVECs migration assay was performed using Boyden chambers with inserts of 8 µm pores (Corning, Bedford, MA, USA). The bottom chambers were filled with 0.75 mL of EndoGRO^TM^-Basal Medium supplemented with 4% FBS. Sub-confluent cells were trypsinized, collected, and resuspended with EndoGRO^TM^-Basal Medium supplemented with 0.1% FBS. Typically, 50,000 cells/0.5 mL, in the presence and absence of test compounds, were added to the top of each migration chamber and cells were allowed to migrate to the underside of the chamber in a humidified incubator at 37 °C and 5% CO_2_ for 8 h. After that, the upper chambers’ non-migrating cells were removed by gently rubbing the area with a cotton swab. The migrating cells were determined using CellTiter-Glo^®^ Luminescent Cell Viability assay (Promega Corporation, Madison, WI, USA) previously described in the cellular viability section.

### 3.11. Vascular Tube Formation Assay

Matrigel Matrix (Corning, Bedford, UK) was thawed, and 40–50 µL was added to the wells of a 96-well plate for coating. In order for the Matrigel to solidify, the plate was kept in a humidified incubator at 37 °C and 5% CO_2_ for 1 h. HUVECs were trypsinized and seeded on the coated plate at a density of 2.5 × 10^4^ cells/100 µL/well in the absence and presence of the indicated low concentrations of butein, frondoside-A, or butein in combination with frondoside-A. After 8 h of incubation, the tube networks at the different wells were photographed using an inverted phase-contrast microscope. The impact of the treatments on the ability of HUVECs to form capillary-like structures was assessed by measuring the total lengths of the formed tubes in the control and drugs-treated wells. Total tube lengths were measured using online image analysis software developed by Wimasis (https://www.wimasis.com/en/products/13/WimTube, accessed on 11 November 2021). The impact of the different treatments on the viability of HUVECs was determined using CellTiter-Glo^®^ Luminescent Cell Viability assay (Promega Corporation, Madison, WI, USA) as previously described in the cellular viability section.

### 3.12. Statistical Analysis

Each experiment was repeated at least three times, and results are expressed as means ± SEM of the indicated data. Statistical analysis was performed with GraphPad Prism7 (La Jolla, CA, USA). The difference between experimental and control values was assessed by ANOVA followed by Dunnett’s multiple comparisons test. For the combination experiments, data were assessed by ANOVA followed by Tukey’s multiple comparisons test. The unpaired t-test was used to assess the difference between two groups. * *p* < 0.05, ** *p* < 0.01, *** *p* < 0.001, and **** *p* < 0.0001 indicate a significant difference.

## 4. Conclusions

In conclusion, we demonstrate that butein decreases lung and breast cancer cell viability and colony growth, leading to a significant decrease in tumor growth in vivo. Butein also decreases cancer cell migration and invasion, suggesting its potential anti-metastatic effect. STAT3 is an oncogene constitutively activated in both A549 and MDA-MB-231 cells used in this study and has been reported to be associated with cancer cell viability/proliferation, migration, and invasion. The reported anti-cancer effects of butein are due, at least in part, to the potent inhibition of STAT3 phosphorylation.

Very few studies have investigated butein anti-cancer effects in combination therapy. In this context, we demonstrate that butein combined with frondoside-A has an additive impact on lung and breast cancer cell viability, colony growth and invasion, and synergistically decreases endothelial cell migration.

This study provides sufficient rationale to carry out pre-clinical research further to confirm the therapeutic potential of this combination therapy using butein and frondoside-A on tumor growth and metastasis in vivo in chick embryo CAM and nude mice tumor xenograft models.

## Data Availability

Not applicable.
